# Ciliated hepatic foregut cyst: report of three cases and review of imaging features

**DOI:** 10.1093/gastro/gov028

**Published:** 2015-06-29

**Authors:** Kianoush Ansari-Gilani, Jamak Modaresi Esfeh

**Affiliations:** 1 Department of Radiology, University Hospitals of Cleveland, Case Western Reserve University, Cleveland, OH, USA; 2 Department of Gastroenterology & Hepatology, Cleveland Clinic, Cleveland, OH, USA

**Keywords:** ciliated hepatic foregut cyst, pseudostratified epithelial cell, magnetic resonance imaging

## Abstract

Ciliated hepatic foregut cysts (CHFCs) are rare cystic lesions which are most commonly asymptomatic. They can be clinically important as they may, on rare occasions, undergo malignant transformation or cause mass effect if significantly enlarged. Three cases of CHFCs are presented in this article and their imaging features are reviewed.

## Introduction

Ciliated hepatic foregut cysts (CHFCs) are very rare hepatic lesions, with less than 100 cases described in the literature since the 19^th^ century [1]. They are important because they can impose diagnostic difficulties and they can, on rare occasions, progress to malignancy [2]. We describe the radiological appearance of three ciliated hepatic foregut cysts, which were confirmed by pathology.

## Case Presentation

### Case 1

**Figure 1.**
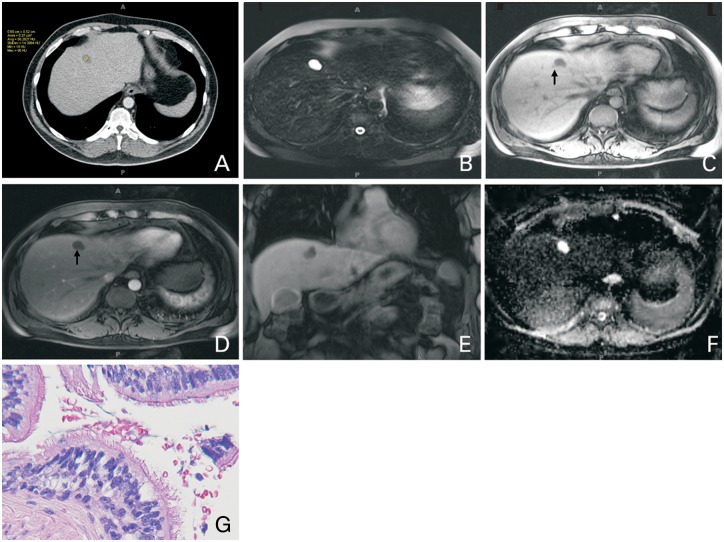
A 52-year-old man with ciliated hepatic foregut cyst. Contrast-enhanced CT (A) shows a hypodense lesion in segment 4 A, with attenuation slightly more than water (56 Hounsfield Unit). The lesion is bright on T2 WI (B), with dependent T1 hyperintense layering (arrows) on pre- (C) and arterial post-contrast (D) images and appears to be bilobal on post-contrast coronal image (E). There is no associated diffusion restriction as can be seen on the Apparent Diffusion Coefficient (ADC) image (F). Biopsy confirmed the presence of ciliated pseudostratified epithelial cells, consistent with CHFC (G).

A 52-year-old man, with history of mixed oligoastrocytoma and right upper quadrant abdominal pain, underwent contrast-enhanced computed tomography (CT) of the abdomen, which showed a 2 cm indeterminate hypoattenuating lesion in segment 4 A (Figure 1

A). The attenuation was slightly more than water attenuation. This was further characterized by contrast-enhanced abdominal magnetic resonance imaging (MRI) (Figure 1B–F), which showed a bilobal 2 cm, T2 hyperintense lesion with no enhancement on post-contrast images and no diffusion restriction. It contained fluid–fluid level and a slightly thickened wall. Based on its location and imaging characteristics on CT and MRI, diagnosis of CHFC was proposed but, given the patient’s underlying malignancy, this was biopsied under ultrasound guidance. Pathology confirmed the presence of a cyst lined by ciliated pseudostratified epithelial cells, consistent with CHFC (Figure 1G).

### Case 2

**Figure 2.**
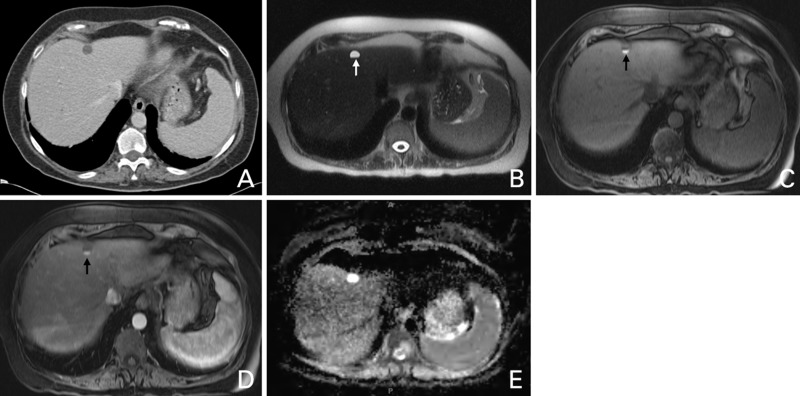
A 62-year-old woman with ciliated hepatic foregut cyst. Contrast-enhanced CT (A) shows a hypodense lesion in segment 4 A, with water attenuation. The lesion is bright on T2 WI, with a dark dependent layer (arrow) (B). The dependent layer is bright on T1 weighted images pre- (C) and on arterial post-contrast (D) images (arrows), suggestive of a fatty or proteinous nature. There is no associated diffusion restriction, as can be seen on the ADC image (E).

A 62-year-old woman with recently diagnosed non-small cell lung cancer underwent CT of the chest, abdomen and pelvis for staging. Abdominal CT showed a 1.5 cm hypoattenuating lesion in segment 4 A (Figure 2

A) which appeared slightly hyperintense on T1 weighted images and demonstrated no enhancement or diffusion restriction (Figure 2B–E). Given its location and imaging findings, the possibility of CHFC was raised and confirmed on biopsy.

### Case 3

**Figure 3.**
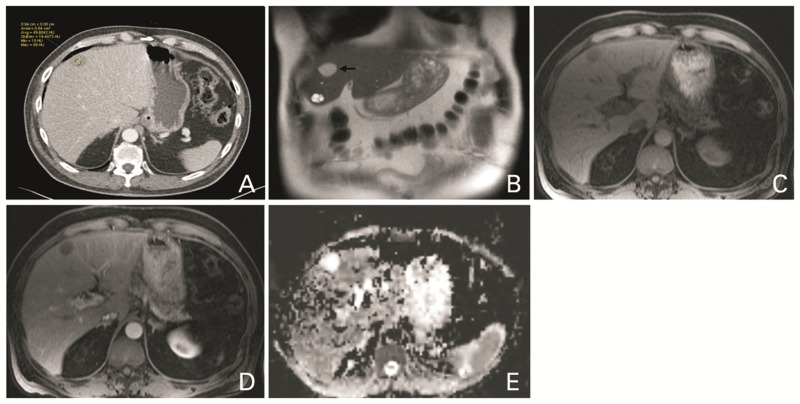
A 68-year-old man with ciliated hepatic foregut cyst. Contrast-enhanced CT (A) shows a hypodense lesion in segment 4B, with attenuation slightly more than water (49 Hounsfield Unit). The lesion (arrow) is bright on T2 WI (B) but not as bright as a cyst also seen in segment 4B. The lesion is hypointense on T1 weighted image (C) with no enhancement after contrast injection (D). There is no associated diffusion restriction, as can be seen on the ADC image (E).

A 68-year-old man with abdominal pain underwent CT of the abdomen, which showed a mass in the lesser curvature of the stomach. On biopsy, this was proven to be a gastrointestinal stromal tumor (GIST). CT also showed a hypoattenuating lesion in segment 4B of the liver, the density of which was slightly higher than water (Figure 3

A). MRI of the abdomen (Figure 3B–E) showed the same lesion, which appeared slightly hyperintense on T1 weighted images and demonstrated no enhancement or diffusion restriction. On T2 weighted images, it appeared less bright when compared with an adjacent multiloculated cyst. Given the image characteristics and its location, the possibility of CHFC was raised. Biopsy was performed to exclude possible metastatic involvement of the liver. Pathology showed a cyst lined by ciliated pseudostratified epithelial cells, consistent with CHFC.

## Discussion

It is difficult to determine the true prevalence of CHFCs because they are generally asymptomatic. They are usually incidental findings on imaging or during surgery [1]. It is suspected that a CHFC is a detached hepatic diverticulum or abnormal tracheobronchiolar bud that may have migrated caudally, to be included with the liver during the early embryonic development of the foregut [3]. They contain a ciliated, pseudostratified columnar epithelium, a layer of subepithelial connective tissue, a smooth muscle layer and outer capsule [4].

CHFCs occur more frequently in men and in the medial segment of the left hepatic lobe (segments 4 A and 4B) [1, 5]. They are unilocular and avascular, usually lying just beneath the anterior surface of the liver in a subcapsular position [6, 7]. The mean size of CHFCs is 3 cm, with a range from 1–12 cm [6, 7].

The appearance under imaging is variable—often attributed to the elements of the cyst contents, including clear serous to white or brown material with different viscosities. On ultrasound, they usually appear as a well-defined anechoic or hypoechoic small masses. They can have water or slightly more than water attenuation on CT [8], but they can also be iso- to hyperdense. On MRI they are T2 hyperintense (usually not as hyperintense as a simple cyst), and have a variable appearance on T1 weighted images [5], including T1 hyper- or hypointensity. They may also show a fluid–fluid layer due to the presence of fatty or protein-rich contents [1].

The differential diagnosis for this lesion includes other unilocular hepatic lesions, such as simple hepatic cyst, echinococcal cyst, epidermoid cyst, pyogenic abscess, intrahepatic choledochal cyst, mesenchymal hamartoma, hypovascular solid tumor, and hepatobiliary cystadenoma or cystadenocarcinoma [5, 9, 10].

There is controversy regarding the management of these lesions. Some believe they are incidental findings and generally asymptomatic, and therefore recommend observation [11], while others recommend a more aggressive approach, such as aspiration or surgical resection, due to a few reported cases of malignant transformation [12]. Given the recent report of malignant transformation [12], serial imaging may be needed for those undergoing cyst sclerosis [13]. The frequency of imaging is, however, not determined.

Overall, most authors agree that CHFCs should be surgically resected for cysts larger than 4–5 cm, lesions that are symptomatic or show interval growth, or asymptomatic lesions with worrying findings on imaging, such as focal wall abnormalities or thick septations [10, 14, 15]. To date, the major risk factor for the malignant transformation of a CHFC is its size. All the above three cases with malignant transformation were larger than 12 cm [13, 15]. There are reports of successful excision of these lesions with minimally invasive (laparascopic) methods [10, 13, 14].

In conclusion, CHFC is a rare hepatic lesion with non- specific but suggestive features on imaging. Although most cases are asymptomatic and found incidentally during imaging for other reasons, they may need follow-up or resection due to rare reported cases of malignant transformation.

*Conflict of interest*: none declared.
